# Monkeypox is an emerging threat to low-middle-income countries amid COVID-19

**DOI:** 10.1016/j.amsu.2022.104344

**Published:** 2022-08-02

**Authors:** Yasmin Jahan

**Affiliations:** a Graduate School of Biomedical and Health Sciences, Hiroshima University, Japan; b Rufaida College of Nursing, Plot #12, 14 Road #02, Block-C, Bachila, Mohammadpur, Dhaka, Bangladesh

The biggest-ever outbreak of monkeypox disease in non-endemic countries started in May 2022 [1]. The World Health Organization (WHO) confirmed the global outbreak of monkeypox has grown to more than 13,069 cases as of July 18, 2022, with 80% in European countries (see Fig. 1) [2]. By coming into intimate contact with lesions, bodily fluids, respiratory droplets, and contaminated materials like bedding, the monkeypox virus can spread from one person to another (see Fig. 2) [1]. A double-stranded DNA virus from the *Orthopoxvirus* genus of the *Poxviridae* family is known as the monkeypox virus. The Congo Basin (Central African) clade and the West African clade are the two clades of the monkeypox virus. The virus was first identified in monkeys in a Danish laboratory in 1958, hence it was named monkeypox [3]. The first known human case was discovered in 1970 in the Democratic Republic of the Congo in a young child. The typical incubation time for monkeypox is between 6 and 13 days, although it can go up to 21 days [4]. In certain people, such as newborns, expectant mothers, or those whose immune systems have been suppressed by other illnesses, monkeypox can be severe, but it usually goes away on its own. Monkeypox spreads from person to person through close contact with someone who has a monkeypox rash, including through face-to-face, skin-to-skin, mouth-to-mouth, or mouth-to-skin contact, including sexual contact. Besides, monkeypox can spread to people when they contact an infected animal (See Fig. 3) [1].

**Fig. 1:**
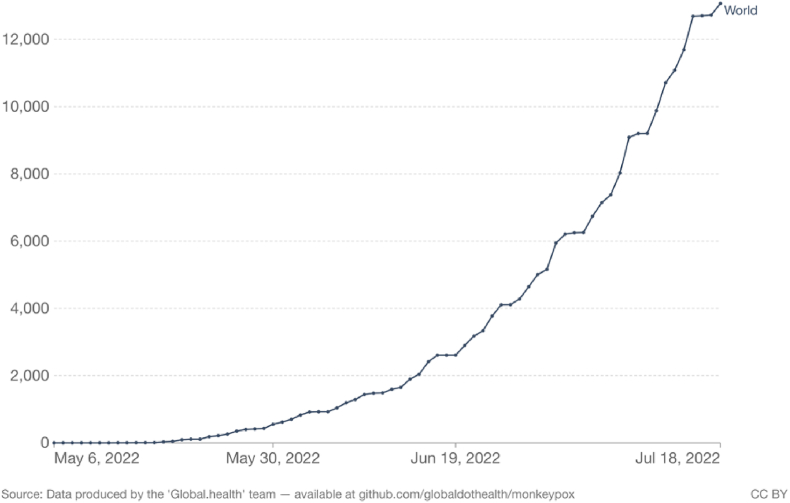
Monkeypox: cumulative confirmed cases globally.

**Fig. 2:**
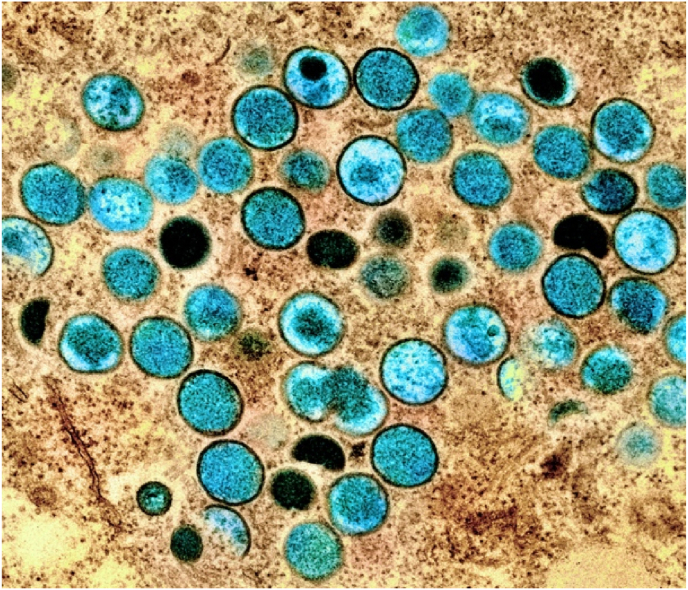
Colorized transmission electron micrograph of monkeypox particles (teal) found within an infected cell (brown), cultured in the laboratory. (Image captured and color-enhanced at the NIAID Integrated Research Facility (IRF) in Fort Detrick, Maryland. Credit: NIAID). (For interpretation of the references to color in this figure legend, the reader is referred to the Web version of this article.)

**Fig. 3:**
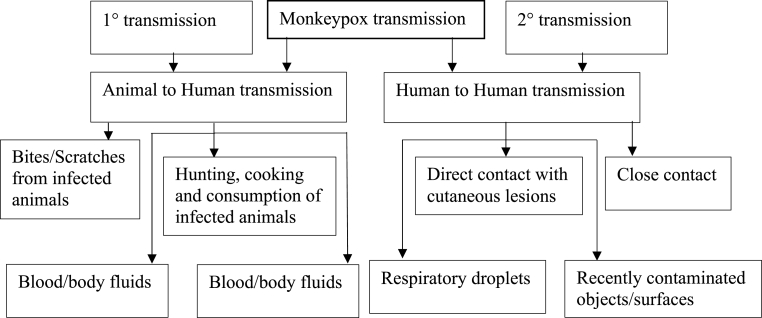
Mode of transmission of monkeypox virus infection.

So far, cases were found in the USA, Canada, Australia, Israel, and several European countries including the UK, Sweden, Spain, Portugal, Netherlands, Italy, Germany, Belgium, and France. Most cases of monkeypox occur in central and western Africa [1]. Human infections in the West African group are less severe than in the Congo Basin group, with case fatality rates appearing to be 3.6% compared to 10.6% in the Congo Basin group. Fortunately, no fatalities have been reported in 2022 so far [1]. However, it is still unclear about the natural history, animal origin, and reservoir host for virus circulation. It may be possible to better understand the zoonotic origin of the virus through intensive surveillance of the monkeypox virus in areas where it is endemic [5]. Therefore, WHO has developed a surveillance system for gathering information, identifying knowledge gaps, and priority research questions for monkeypox research in order to provide an action plan that can be quickly implemented to control the outbreaks [1].

On May 22, the Bangladeshi government announced a health alert amid the outbreak of the monkeypox virus and noted that no one has yet been infected with the monkeypox virus in Bangladesh. However, it is almost certain that the virus will spread throughout the country. A number of preventative actions must be taken to stop the COVID-19 pandemic from further decimating Bangladesh's already-struggling healthcare system [6]. The COVID-19 epidemic presented a number of challenges for Bangladesh's healthcare system because of its fragile infrastructure and a shortage of skilled workers. It was extremely challenging for hospitals and healthcare organizations in Bangladesh to ensure the availability of sufficient staff, ventilators, hospital beds, medical specialists, and laboratory equipment [7]. Therefore, to prevent a COVID-19 or monkeypox-like outbreak in Bangladesh, the appropriate health authorities must take a proactive approach and launch widespread awareness campaigns that stress the significance of hygiene practices, self-quarantining, and other pertinent safety precautions.

In order to address this issue in limited resources countries like Bangladesh, doctors must be sufficiently knowledgeable about the disease's presenting signs and symptoms to ensure that suspected patients are promptly quarantined rather than simply receiving symptomatic treatments. In order to stop the spread of the contagious virus, hospitals should also have well-equipped isolation units ready to place patients under quarantine right away. Additionally, effective surveillance systems must be created in order to track cases and respond appropriately. Public education sessions on the atypical pox rash and its progressive stages, which include atypical macules, papules, vesicles, pustules, and scabs, are among the preventive actions that can help in dealing with any suspected case before it spreads.

Monkeypox has no known cure, but according to health professionals worldwide, the smallpox vaccine is 85% effective in preventing illness. However, epidemiological studies, transmission dynamics, and the ecology of the disease are still not fully understood and need further focus. The animal reservoir is yet unclear, and case management and effective outbreak response strategies are poorly documented. Up-to-date complete, consistent and longer-term research is sorely needed to inform and guide evidence-based response and management of monkeypox outbreaks.

## Ethical approval

Not applicable.

## Sources of funding

None.

## Author contribution

I, Yasmin Jahan have participated in the study concept or design, literature search, produced a table, and wrote the final manuscript of the paper.

## Declaration of competing interest

The author declares that there is no conflict of interest.

## Registration of research studies

1. Name of the registry: Not applicable
2. Unique Identifying number or registration ID: Not applicable
3. Hyperlink to your specific registration (must be publicly accessible and will be checked): Not applicable

## Guarantor

Yasmin Jahan.

## Provenance and peer review

Not commissioned, externally peer-reviewed.

## Consent

Not applicable.
